# Nanoformulations of α-Mangostin for Cancer Drug Delivery System

**DOI:** 10.3390/pharmaceutics13121993

**Published:** 2021-11-24

**Authors:** Lisna Meylina, Muchtaridi Muchtaridi, I Made Joni, Ahmed Fouad Abdelwahab Mohammed, Nasrul Wathoni

**Affiliations:** 1 Department of Pharmaceutics and Pharmaceutical Technology, Faculty of Pharmacy, Universitas Padjadjaran, Sumedang 45363, Indonesia; lisna@farmasi.unmul.ac.id; 2 Department of Pharmaceutics and Pharmaceutical Technology, Faculty of Pharmacy, Universitas Mulawarman, Samarinda 75119, Indonesia; 3 Department of Pharmaceutical Analysis and Medicinal Chemistry, Faculty of Pharmacy, Universitas Padjadjaran, Sumedang 45363, Indonesia; muchtaridi@unpad.ac.id; 4 Department of Physics, Faculty of Mathematics and Natural Sciences, Universitas Padjadjaran, Sumedang 45363, Indonesia; imadejoni@phys.unpad.ac.id; 5 Functional Nano Powder University Center of Excellence, Universitas Padjadjaran, Sumedang 45363, Indonesia; 6 Department of Pharmaceutics, Faculty of Pharmacy, Minia University, Minia 61519, Egypt; ahmed.mohamed1@minia.edu.eg

**Keywords:** *Garcinia mangostana* L., nanotechnology, drug delivery, cancer therapy

## Abstract

Natural compounds are emerging as effective agents for the treatment of malignant diseases. The active constituent of α-mangostin from the pericarp of *Garcinia mangostana* L. has earned significant interest as a plant base compound with anticancer properties. Despite α-mangostin’s superior properties as an anticancer agent, its applications are limited due to its poor solubility and physicochemical stability, rapid systemic clearance, and low cellular uptake. Our review aimed to summarize and discuss the nanoparticle formulations of α-mangostin for cancer drug delivery systems from published papers recorded in Scopus, PubMed, and Google Scholar. We investigated various types of α-mangostin nanoformulations to improve its anticancer efficacy by improving bioavailability, cellular uptake, and localization to specific areas These nanoformulations include nanofibers, lipid carrier nanostructures, solid lipid nanoparticles, polymeric nanoparticles, nanomicelles, liposomes, and gold nanoparticles. Notably, polymeric nanoparticles and nanomicelles can increase the accumulation of α-mangostin into tumors and inhibit tumor growth in vivo. In addition, polymeric nanoparticles with the addition of target ligands can increase the cellular uptake of α-mangostin. In conclusion, nanoformulations of α-mangostin are a promising tool to enhance the cellular uptake, accumulation in cancer cells, and the efficacy of α-mangostin as a candidate for anticancer drugs.

## 1. Introduction

Cancer is the second leading cause of death globally and was responsible for an estimated 9.6 million worldwide deaths in 2018 [1]. The incidence of cancer cases is gradually increasing because of population growth and life expectancy with a projected increase in cases of up to 75% and is predicted to be the main cause of death by 2030 [2,3,4]. Currently, cancer treatments are surgery, radiotherapy, and anticancer drugs (chemotherapy and immunotherapy). Surgery and radiotherapy are most effective for the treatment of local and non-metastatic cancers, but less efficient for metastatic cancers [5,6]. Furthermore, anticancer drugs are the current choice for the treatment of metastatic cancer, because they can reach every organ in the body through the bloodstream [7]. However, anticancer drugs such as chemotherapy have many limitations, including the high incidence of side effects, limited effectiveness, multidrug resistance, and is highly toxic to growing healthy cells due to their non-specific targeting of cancer cells [8,9].

The development of natural product-based compounds is a promising strategy for cancer treatment. Various plant-based molecules such as α-mangostin, curcumin, and taxanes have chemopreventive or anticancer properties based on in vitro and in vivo studies [10,11,12,13,14,15,16,17,18,19,20,21]. α-mangostin is a xanthone derivate extracted from the skin of the fruit of *Garcinia mangostana* Linn known as the queen of fruits. It has revealed several anticancer properties [22,23,24,25,26] in various kinds of cancers such as colon [27,28], lung [29], pancreas [30], breast [22,31], skin [32], and blood [33]. However, α-mangostin has some physicochemical properties drawback, especially limited water solubility, resulting in poor absorption and low bioavailability on intravenous and oral administration, which can affect its effectiveness as an anticancer therapeutic agent [34,35,36].

Thus, to overcome those limitations, nanoparticles or nano-structured materials were introduced as a drug delivery system as an alternative to conventional drug delivery systems to achieve high water solubility and specific biodistribution [37,38]. Various nanotechnology-based drug delivery systems such as polymeric nanoparticles, solid lipid nanoparticles, liposomes, and nanomicelles have been designed to improve the low-water solubility of α-mangostin [39]. Many researchers reported that these formulations can enhance the therapeutic efficacy achieved from enhanced targeted localization and improved cellular internalization of α-mangostin nanoformulation in cancer cells. Despite many developments in nanoformulation of drug delivery systems for α-mangostin, the engineering on morphology and chemical structure related to their targeted or selective recognition of cancer cells and control released drug delivery system remain challenges. This review highlights various nanotechnological approaches used for α-mangostin delivery focused on cancer therapy.

## 2. Methodology

**Figure 1:**
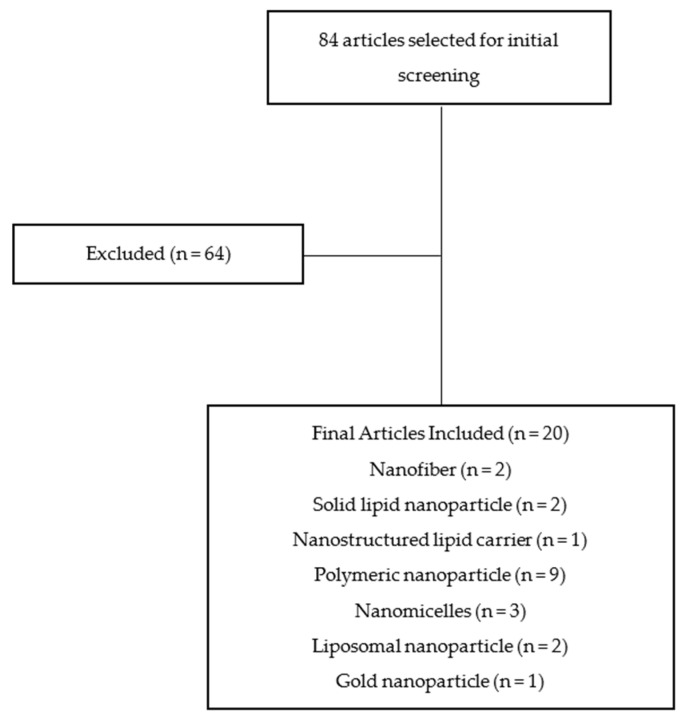
Flowchart of methodology.

**Figure 2:**
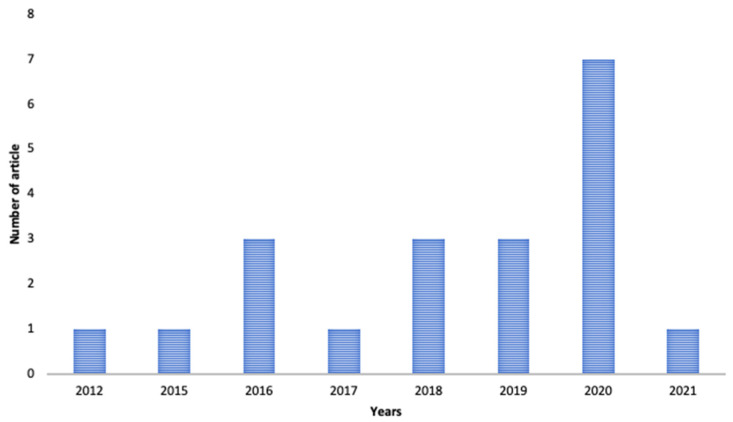
Distribution of articles based on the year of publication nanoformulation of α-mangostin for cancer.

This review is based on the literature obtained from Google Scholar, PubMed, and Scopus using the keywords “nanoformulation of α-mangostin for cancer drug delivery system”, “nanoparticle formulation of α-mangostin for cancer drug delivery system”, and “α-mangostin nanoparticle for cancer drug delivery system” published in the last 10 years. Opinions, assessments, and unrelated subjects such as pharmacological characteristics and bioactivities were utilized as exclusion criteria. The flowchart of the methodology is shown in Figure 1. The distribution of articles based on the year of publication can be seen in Figure 2.

## 3. α-Mangostin

**Figure 3:**
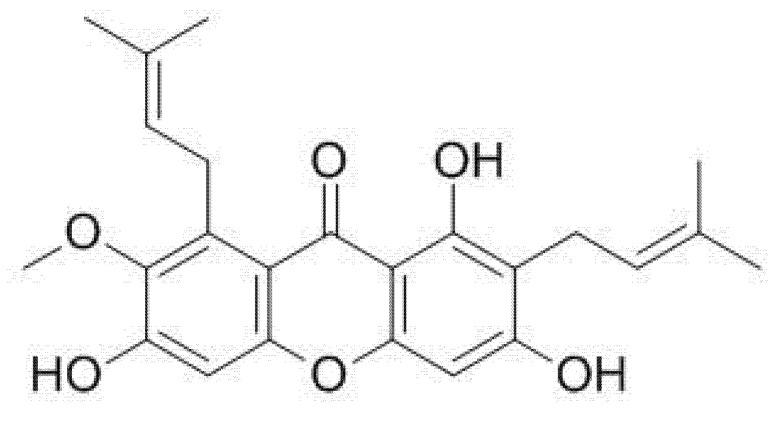
Chemical structure of α-mangostin.

α-mangostin (Figure 3) is a metabolite of 1,3,6,7-tetrahydroxy-2,8-di(3-methyl-2-butenyl) xanthones isolated from mangosteen pericarps [23,40,41,42]. α-mangostin is soluble in methanol and has a water solubility of 2.03 × 10^−4^ mg/L at 25 °C (Table 1) [43,44]. The low solubility of α-mangostin in water causes low bioavailability. Pharmacokinetic studies performed in mice after a single oral dose (20 mg/kg) showed low bioavailability (F = 2.29%) which was thought to be due to low gastrointestinal absorption and rapid metabolism of α-mangostin in the liver and small intestine [45].

α-mangostin has been found to possess a wide range of biological activities such as anticancer [22,27,46,47,48,49], antioxidant [50], antibacterial [51,52], hepatoprotective [53], cardioprotective [54], antimalarial [55], anti-obesity activities [56], and neuroprotective properties in Alzheimer’s disease [57]. Previous studies have shown that α-mangostin acts against cancer cells through several mechanism pathways such as suppressing fatty acid synthase [31], downregulating the PI3K/Akt pathway [46], and β-catenin gene regulation [28]. Despite having the above-mentioned anticancer properties, its poor oral bioavailability remains the main drawback, limiting its clinical potential. As a result, these issues have led to the development of α-mangostin nanoformulations in the quest of improving α-mangostin delivery for better therapeutic outcomes.

## 4. α-Mangostin Nanoformulation

The application of nanotechnology to medicine has resulted in the development of nanoparticle therapeutic carriers [59]. The recommended particle size for cancer treatment is 10–200 nm, which allows it to easily infiltrate tumor blood vessels that leak and collect in tumor tissue, reducing side effects [60].

Several types of nanoparticles are in different stages of the development process as cancer drug delivery systems, including nano polymers, micelles, liposomes, lipid-based carriers (lipid emulsions and lipid–drug conjugates), and several ligand-targeted products (such as an antibody, folate, and transferrin conjugated molecules) [59,61,62,63]. Nanoparticles usually consist of two or more components, and at least one of them is an active pharmaceutical ingredient. The materials and methods selection for nanoparticle assembly is mainly based on the size and shape of the nanoparticles, physicochemical properties of the active ingredient, the target delivery, and their stability and safety. Components and methods selected are very crucial to ensure the intended administration method and drug targeting abilities [64].

**Figure 4:**
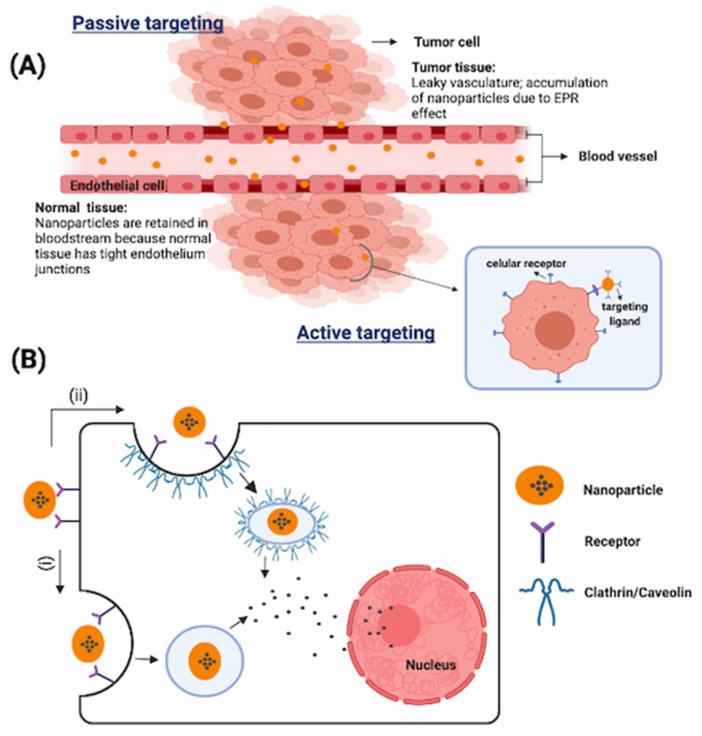
(**A**) Nanoparticles are intended to exploit the enhanced permeability and retention effect to exit the blood vessels through leaky vasculature, accumulate within tumor tissues, and enter the cells via endocytosis before releasing their ‘drug’. Conversely, owing to the tight endothelium junctions in normal tissues, the nanoparticles would remain in the bloodstream. (**B**) Small nanoparticles could be internalized through many pathways such as (i) clathrin or caveolin-mediated endocytosis and (ii) clathrin/caveolin-independent endocytosis.

**Figure 5:**
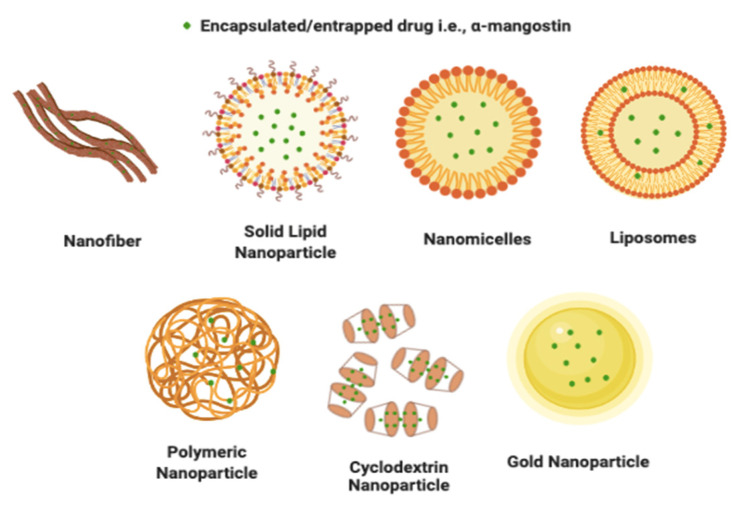
Nanoformulation of α-mangostin for cancer drug delivery.

Nano-based cancer treatment, which employs a mixture of nanomaterials and chemotherapeutic drugs, plays a significant role in the therapeutic effects against cancer [60,65,66]. Nanoparticles preferentially accumulate in tumors due to their enhanced permeability and retention effect (EPR) (Figure 4), resulting in differences in the biodistribution between conventional chemotherapeutics and nanoparticle drug carriers, where nanoparticle-based chemotherapy can achieve higher drug concentrations in the intratumor environment, and eventually better therapeutic efficiency and lower toxicity [67,68,69]. Various nanoformulations are being explored to enhance the delivery of α-mangostin to tumor sites. α-mangostin nanoformulations for cancer should enhance anticancer activity and selectivity compared to free α-mangostin, and at the same time be non-toxic to normal cells. α-mangostin nanoformulations for cancer that have been reported in the literature include nanofibers, nanostructures lipid carrier, solid lipid nanoparticles, polymeric nanoparticles, nanomicelles, liposomes, and gold nanoparticles. These nanoformulations are described in Figure 5 and summarized in Table 2.

### 4.1. Nanofibers

Nanofibers are one-dimensional nanomaterial with a size of less than 500 nm [85]. Currently, nanofibrous polymer materials are being thoroughly investigated for a variety of medical uses. The use of nanofiber system drug carriers in anticancer therapy offers numerous benefits, including the ability to form fibers with varying diameters ranging from nanometers to sub-microns, surface modification, having a large outer surface ratio and unique porosity to maximize the drug encapsulation process, controlled and sustained drug release in the desired workplace to improve efficacy [86,87,88,89,90,91].

In 2019, D.T. Pham et al., tried to develop a fibroin nanoformulation for α-mangostin using a natural polymer (silk fibroin) as a carrier, EDC or PEI as a crosslinker, and utilize the desolvation method. Fibroin is a natural biocompatible and biodegradable protein extracted from *Bombyx mori* silk. This material has been used as a carrier for anticancer drugs [92,93]. The observed mean size was around 300 nm with a narrow size distribution with PI < 0.3 and zeta potentials from −15 to +30 mV proportionally to the increase of EDC content. The formula that uses a crosslinker had a higher entrapment efficiency and loading capacity (70%) and (7%) than the formula without a crosslinker (<50% and <5%). Compared to the free α-mangostin, nanoparticles increased the drug’s solubility up to threefold. In vitro cytotoxicity of α-mangostin and α-mangostin loaded nanoparticles was conducted in Caco-2 and MCF-7 cells. The results showed that the nanoparticle maintained the apoptotic effect of α-mangostin and exhibited improved cytotoxicity (i.e., lower IC_50_) than the free α-mangostin. It is related to differences in the cellular uptake mechanisms. Free α-mangostin in solution penetrates the cells via a passive transport pathway, limited by cell lipid bilayers and the efflux pumps. Whereas, silk fibroin nanoparticles loaded with α-mangostin effectively enter the cell via surface adsorption and endocytosis pathways, where it is possible that two amino acids located near the N-terminal of the fibroin heavy chain can bind to surface receptor integrin. In particular, the cell surface receptor integrin was overexpressed in many cancer cells, including colon and breast cancer. This binding will induce the endocytosis process. Therefore, the formulation of α-mangostin using fibroin nanoparticles can enhance IC_50_ so that it can boost the effectiveness of α-mangostin as a cancer medicine [3].

Taokaew et al. formulated bacterial cellulosic nanofiber film synthesized from *Acetobacter xylinum* (Gram-negative aerobic bacteria). This bacterial-based nanofiber has several advantages, such as its similarity to the natural extracellular matrix, high loading capacity, non-toxicity, and resistance to heating during the sterilization process. Bacterial cellulosic cytotoxicity testing was performed on cancer cells (B16F10 melanoma and MCF-7 breast cancer cells) and normal cells (HaCat fibroblast and hGF keratinocyte cells). The loading capacity of α-mangostin was up to 7.33% and the loading efficiency ~67.3% with a concentration proportion of 0.01–1% (2, 24, and 250 mg/cm^3^). The characteristics of bacterial cellulosic nanofiber are cellulose fibers with a diameter of 50–100 nm with an empty cavity that stretches between the fibers to absorb the active ingredients. The cytotoxicity of nanofibers on cancer and normal cells was tested at three α-mangostin loading concentrations, namely 2, 24, and 250 mg/cm^3^. The findings revealed that a concentration of 2 mg/cm^3^ had no cytotoxic effect on B16F10 melanoma, but a higher concentration of α-mangostin inhibited growth and caused changes in cell morphology that suggested membrane damage in B16F10 melanoma and MCF-7 breast cancer cells. Furthermore, testing on normal cells showed a decrease in viability, namely ~40% in hGF cells and 38% in HaCaT cells during 24 h of observation. After incubation for 48 h, HaCaT cells experienced an increase the viability by 111% while hGF cells by 53%. It suggests that the α-mangostin nanofiber is slightly more toxic to HaCaT cells than hGF cells. It was concluded that bacterial cellulosic nanofiber α-mangostin has lower cytotoxicity against normal cells compared to cancer cells [70].

### 4.2. Solid Lipid Nanoparticle

Solid lipid nanoparticles (SLN) are colloidal particles constructed from biodegradable physiological lipids which are solid at room and body temperature. It has sizes ranging from 50 to 1000 nm according to the manufacturing method and type of lipid used. SLN is widely used as a carrier for cancer medicines because it has many benefits, such as solvent-free preparation, being composed of biocompatible and biodegradable materials, site-specific targeting, physical stability, and controlled release for both hydrophilic and lipophilic drugs [94,95,96].

F. Bonafè et al. attempted to develop solid lipid nanoparticles of α-mangostin conjugated with CD44 thioaptamer as a ligand targeting multicellular tumor spheroids (MCTSs) by MCF-7 cell. Using a nanoprecipitation technique, lipid nanoparticles were synthesized from PLGA, soybean lecithin, and DSPE–PEG2000–COOH, and the thioaptamer was conjugated to nanoparticles using the two catalysts, 1-ethyl-3-(3-dimethylaminopropyl) carbodiimide, and N-hydroxysuccinimide [71]. PLGA deployed as the core can carry hydrophobic drugs with high loading capacity. Then, hydrophilic coating by soybean lecithin and DSPE-PEG2000-COOH provides steric protection capable of reducing systemic clearance rates, prolonged circulation half-life in vivo, and functional groups for surface modification attachment of ligands [97]. The mean diameters of the nanoparticles were 227.0 ± 88 and 174.0 ± 29 nm (after filtered by 200 nm cut-off). α-mangostin nanoparticles (0.1 μg/mL) induced significant dissociation of MCTSs at a dose 10 times lower than the α-mangostin (1 μg/mL). These results suggested that nanocarrier is a suitable vehicle for α-mangostin to suppress tumors at low concentrations. Moreover, the nanoparticles conjugated to the CD44 thioaptamer could reduce the size of the spheroids. It suggests that several cells died or slowed down their process of duplication [71]. Another solid lipid nanoparticle was prepared by V. Kumar and tested against diethylnitrosamine-induced hepatocellular cancer. This method generated particles with a size of 182.3 nm and a polydispersity index of 0.203, and it reduced hepatic nodules by 84.5% and 93.4%, respectively. The nanoparticle α-mangostin controlled the PI3K and Akt pathways, which are involved in the inhibition of hepatic cancer growth and proliferation, as well as its chemoprotective action [98].

### 4.3. Nanostructured Lipid Carriers

Nanostructured lipid carriers are novel therapeutic formulations consisting of physiological and biocompatible lipids, surfactants, and co-surfactants. The use of NCL as a carrier for chemotherapy agents is very promising because it is a bio-compatible and bio-degradable lipid-based nanoparticle that is able to improve its physical and chemical stability, and significantly increase the therapeutic capacity with low pharmacokinetic properties [99,100,101].

The mucoadhesive NLC was developed for the possible oral delivery of α-mangostin. Nanostructure lipid carrier coated with oleoyl-quaternized-chitosan (NLC-CS) with high-pressure homogenization process was used for the nanoparticles prepared in a range of nanoparticle sizes from 200 to 400 nm, low polydispersity, zeta potentials 40.9 mV, with excellent encapsulation efficiency (>90%). Results demonstrated that the NLC-CS has a higher toxicity than the NLC against Hela and Caco-2 cells. The CS-NLC particles resulted in better cellular uptake than the NLC particles due to the mucoadhesive properties of the CS-NLC particles. Positively charged nanoparticles have a higher internalization rate than neutral or negatively charged species. This behavior is due to the strong electrostatic adhesion of positively charged chitosan to negatively charged mucosal surfaces. Furthermore, CS-NLC outperformed NLC due to improved mucoadhesion of the particles with the cells, allowing for internalization. These findings support the use of surface-modified CS-NLC nanoparticles as mucoadhesive carriers for drugs to cancer cells [72].

### 4.4. Polymeric Nanoparticle

Polymeric nanoparticles are particles in the size range from 1 to 1000 nm and can be loaded with active compounds trapped in or surface adsorbed onto the polymer core [102]. These systems are typically structured through spontaneous assembly in which the therapeutic compound is trapped within the core of the nanoparticle structure [103]. Generally, polymeric nanoparticles are composed of natural polymers (chitosan and alginate) and synthetic polymers (PLGA and cyclodextrins) [104]. There are many advantages to using polymeric nanoparticles for the delivery of anticancer drugs in cancer treatment, among others, they can be used to deliver various types of drugs such as hydrophilic and hydrophobic drugs, peptides, and biological macromolecules via several routes of administration [105], improve the drug solubility [106], provide controlled release, increase bioavailability, therapeutic index, can transport active ingredients to targeted tissues or organs at a specified concentration [102,107], surface modification with ligand linking for stealth and targeted drug delivery, biocompatibility, biodegradability, and low toxicity [108].

#### 4.4.1. PLGA Nanoparticle

Verma et al. developed α-mangostin encapsulated PLGA nanoparticles to improve the bioactivities of α-mangostin for inhibiting pancreatic cancer stem cells (Pan CSCs) in human and KC mice (PdxCre; LSL-KrasG12D) (Pan CSC). This formula produces a particle size of 186.3 ± 6.42 nm, a zeta potential of 0.03 ± 0.005 mV, and a drug encapsulation of 51.16 ± 2.61%. The results indicated that α-mangostin nanoparticles inhibited cell proliferation of Pan CSCs and pancreatic cancer cell lines more effectively than α-mangostin and had no significant effect on human pancreatic normal ductal epithelial [35]. In another study, V. Chandra Boinpelly et al. reported that α-mangostin-encapsulated PLGA nanoparticles inhibit human colorectal cancer cells and colony deformation in colorectal cancer HCT116 and HT29 cells in a dose-dependent manner at a dose of 0–10 mol/L [73]. These findings suggest that α-mangostin encapsulated PLGA nanoparticles are appropriate for use as carriers to advance the effectiveness and therapeutic effects of α-mangostin for pancreatic cancer and colorectal cancer.

#### 4.4.2. PEG-PLA Nanoparticles

In 2020, J. Feng et al. developed cancer-associated fibroblasts (CAFs) targeting polymer nanoparticle (methoxy poly (ethylene glycol)3000-poly (lactic acid)34000 (Me-PEG-PLA) and maleimide-poly (ethylene glycol)3000-poly (lactic acid)34000 (Male-PEG-PLA) coated with CREKA peptide and loaded with α-mangostin. The concept of using CAFs-targeting nanoformulation α-mangostin is an effective method of modifying tumor environment to enhance pancreatic ductal adenocarcinoma chemotherapy. Nanoparticles with spherical shape particles and average particle size diameter of α-mangostin nanoparticles [NP(α-M)] is 103.76 ± 7.45 nm; α-mangostin nanoparticles coated with CREKA peptide [CRE-NP(α-M)] is 106.93 ± 3.69 nm with a zeta potential of −31.77 ± 2.12 mV, respectively. The negative charge generated by the nanoparticles renders an optimal possibility to develop the EPR effect when examined in vivo. The nanoparticles effectively reduced the production of secreted mass extracellular matrix both in vitro (NIH3T3 cells) (5.8-fold) and in vivo by blocking the TGF-β signaling pathway and significantly inhibiting the tumor growth (<70%), inducing apoptosis and necrosis on an orthotopic mice model. Biodegradable nanocarriers (PEG-PLA) demonstrate controlled and continuous release behavior. Additionally, the use of PEG can avoid the elimination of nanoparticles by the reticuloendothelial system, which further increases the circulation time in the body. Moreover, CREKA peptide as a target ligand can bind to the fibroin–fibronectin complex that is overexpressed in the tumor mass extracellular matrix, thereby increasing the accumulation in the tumor region. From the results achieved, we can infer that this formula could be amplified as an efficient method to enhance the therapeutic effects [78].

#### 4.4.3. Chitosan-Alginate Nanoparticles

Samprasit et al. formulated α-mangostin-loaded chitosan/alginate (CS/ALG) nanoparticles cross-linked with genipin (GP), used the ionotropic gelation method, and evaluated antitumor activity against the colorectal cancer cells (HT-29) [77]. Chitosan is composed of amino groups with a polycationic charge that interacts with negatively charged polymers such as alginate, forming colloid nanoparticles. Chitosan and alginate complexes have attracted attention due to their easy processing [109,110,111]. Genipin acts as a cross-linking agent that reacts with the primary amine group of chitosan to form a rigid nanoparticle structure with a slow degradation rate, making it capable of controlling swelling, degradation, and drug release [111]. The mean particle sizes of GP nanoparticles and non-GP nanoparticles were 477.2 ± 32.2 and 437.6 ± 50.3 nm. The percentage of loading efficiency and capacity of GP nanoparticles is three times higher than that of non-GP nanoparticles. It demonstrates that GP enhanced the α-mangostin loading, wherein during the cross-linking process α-mangostin may be trapped within the GP nanoparticles, resulting in increased loading of α-mangostin. It is also in line with the increase in the particle size of the GP nanoparticles. In vitro release tests simulated the state of the digestive tract at pH 1.2, 6.8, and 7.4. The result is that the release rate of α-mangostin is slower than the non-GP nanoparticles at all pH conditions. Genipin can control the degradation, diffusion, erosion of the nanoparticles, thereby inhibiting α-mangostin release into the medium. From the entire pH range tested, the release of α-mangostin at low pH was significantly lower than at high pH. At high pH, the amine group of chitosan undergoes deprotonation and loses its charge, causing weak electrostatic interaction between chitosan and alginate and unstable nanoparticles, which implies a higher release of α-mangostin. The pattern of slow release of α-mangostin from GP nanoparticles in acid medium and rapid release of α-mangostin at higher pH illustrates that GP nanoparticles can concentrate on the release of α-mangostin in the small intestine and colon [77,111,112].

The viability of the cells treated with varying concentrations of blank GP nanoparticles showed no substantial decrease in cell viability. As a result, blank GP nanoparticles have little effect on cell viability and protected drug carrier. The cytotoxicity of the α-mangostin-loaded GP nanoparticles was dose-dependent. Cell viability was lower at concentrations of 200 and 300 μg/mL of α-mangostin-loaded GP nanoparticles compared to the control. These findings are associated with α-mangostin’s cytotoxicity, confirming that α-mangostin-loaded nanoparticles and α-mangostin have antitumor action against colorectal adenocarcinoma cells. It concluded that chitosan/alginate nanoparticles cross-linked with genipin could be promising candidates for a controlled release drug delivery system of α-mangostin to the large intestine [77].

#### 4.4.4. Chitosan-Kappa Carrageenan Nanoparticles

Wathoni et al. developed an enteric-coated nanoparticle of α-mangostin-loaded chitosan-kappa carrageenan. Kappa carrageenan is a natural polymer used as an encapsulator that will protect nanoparticles from gastric acid because of its low sensitivity to pH and ionic strength. This formula yields an average particle size of 200–400 nm, high entrapment efficiency, and increased solubility. In vitro release testing showed that the nanoparticles produced an initial burst release for pH 1.2 and 7.4, respectively, followed by a slow and sustained release. Cytotoxicity in MCF-7 cells showed an increase in nanoparticle activity compared to free α-mangostin. The IC_50_ of α-mangostin, α-mangostin-chitosan nanoparticle, and α-mangostin-chitosan-kappa carrageenan was 8.2, 6.7, and 4.7 g/mL, respectively [79].

### 4.5. Cyclodextrin Nanoparticles

Cyclodextrins are non-reducing oligosaccharides of starch-modified products with a ring-shaped chemical structure and are formed through the cyclization process by CGTase activity (cyclodextrin glycosyltransferase). Suitable reaction conditions will produce three main groups of cyclodextrin: α-, β-, and γ-cyclodextrins consisting of 6, 7, and 8 units of (1,4) linked d(+)-glucopyranose. As there are numerous hydroxyl groups linked to the top and bottom of the molecule, it is water-soluble, but the inside of the cyclic structure is hydrophobic and may trap hydrophobic molecules; this configuration is known as the “molecular pocket”. The number of glucose units determines the cavity size, and the cavity diameters of α-, β-, and γ-cyclodextrin are 5.7, 7.8, and 9.5 Å, respectively [113,114,115]. The cyclodextrins molecular pocket is used to trap hydrophobic medicines like α-mangostin without interfering with their bioactivity [74].

Cyclodextrin-based nanoparticles (CDNP) encapsulating α-mangostin were developed by a polyaddition reaction using epichlorohydrin and examined cytotoxicity in CT26WT cancer cells. The cyclodextrin-containing hyperbranched polymer was produced by a poly-addition reaction between cyclodextrin and epichlorohydrin (ECH) that capably integrates numerous cyclodextrins into the polymer using a relatively easy synthesis technique. The results showed that βCDNP showed a higher loading ratio of α-mangostin. The cell viability results after 24 and 48 h of incubation indicated the CDNP themselves are inert, while in the case of CDNP α-mangostin complexes, the cell viability is different depending on the type of CD. The αCDNP/α-mangostin and γCDNP/α-mangostin showed high cytotoxicity for cells after 24 h. However, cell viability decreased significantly for βCDNP/α-mangostin after 48 h. It indicates that the α-mangostin in the αCDNP and γCDNP systems can be easily released from the polymer network structure so that almost no cells survived after 24 h of incubation. In contrast, because βCDNP can strongly resist α-mangostin release, the βCDNP system does not show any toxicity after 24 h. However, due to the presence of other candidates of the guest molecule for βCD, such as cholesterol in the cell membrane, the α-mangostin in βCDNP was probably gradually exchanged by the guest molecules, resulting in releasing α-mangostin and inducing higher toxicity after 48 h [74]. Continuing the previous study, V.T.H.Doan et al. focused on the anticancer activity of CDNP/α-mangostin. They have evaluated in vitro and in vivo anticancer efficacy using a CT26WT cell line. There was an increase in the IC50 value from CDNP compared to α-mangostin in the monolayer culture, in the order of α-mangostin (~14.5 μM), αCDNP/α-mangostin (~27.7 μM), ɣCDNP/α-mangostin (~43.5 μM), and βCDNP/α-mangostin (~50.4 μM). It occurs due to nanoparticles retention capability for α-mangostin release from the system. In this study, they examined the in vitro cytotoxicity of α-mangostin and CDNP/α-mangostin on CT26WT spheroid cells. The IC50 values increased ~33 times in α-mangostin and ~9 times in CDNPs/α-mangostin. In the spheroid cells, the drug must penetrate the spheroid cellular layer to migrate inside and kill the cells. This is because of the hydrophobicity of α-mangostin that was easily adsorbed on the spheroid cells and causes a decrease in drug concentration in the spheroid. Whereas, CDNP reduces the hydrophobicity of α-mangostin so that it has better cellular penetration than α-mangostin, which has better cytotoxicity and lower IC50 than that observed for α-mangostin. The contrast in IC50 values between βCDNP/α-mangostin and ɣCDNP/α-mangostin is due to the higher α-mangostin retention capability of βCDNP than γCDNP. α-mangostin may not be released much in the case of βCDNP/α-mangostin, appearing in a higher IC_50_ than γCDNP/α-mangostin. It is relevant to the cavity size of CDs; γCDNP/α-mangostin have a larger cavity than βCDNP/α-mangostin. Impressively, in vivo anticancer efficacy showed changes in the tumor volume and tumor growth ratio after i.v. (10 mg/kg) of α-mangostin and βCDNP/α-mangostin into BALB/c mice bearing CT26WT tumors. α-mangostin showed tumor volume and tumor growth ratio gradually increased over 22 days and reached 1145 ± 194 mm^3^ in tumor volume and tumor growth, indicating an increase of ~10-fold. While, the βCDNP/α-mangostin showed appreciable suppression of tumor growth. The tumor volume was 501 ± 372 mm^3^ and the tumor growth ratio was ~4 [75]. The increase in tumor size and volume on α-mangostin treatment was due to α-mangostin swiftly clearing from the blood within 3.5 h after intravenous injection with a single dose [36]. Consequently, to achieve significant anticancer efficacy a frequent α-mangostin administration is required. Whereas for βCDNP/α-mangostin, most of the particles remained in the serum after 6 h, showing good circulation in the blood, which may be due to the hydrophilic surface of βCDNP. Biodistribution testing showed βCDNP appeared to accumulate in the tumor [75].

In another study, M.P. Nguyen Thi et al. synthesized ßCDNP/α-mangostin with particles size of <50 nm and zeta potential of −38 mV. Cytotoxicity against lung cancer cells A549 demonstrated that βCDNP/α-mangostin exhibited lower IC_50_ values than α-mangostin (2.34 and 4.86 g/mL, respectively). Furthermore, fluorescence microscopy revealed that βCDNP/α-mangostin is carried into cancer cells and changes the shape of the cell’s nucleus. These findings suggest that βCDNP/α-mangostin improves bioavailability and anticancer efficacy [76].

### 4.6. Nanomicelles

Nanomicelles are colloidal nano-sized structures with a hydrophobic core and a hydrophilic shell [116]. Nanomicelles have been widely used as the delivery system for anticancer drugs, in particular for the delivery of water-insoluble bioactive compounds [117]. Micelles structures formed amphiphilic block copolymers containing hydrophilic blocks and hydrophobic blocks, some hydrophilic and nonionic polymers, such as PEG, poly (N-vinyl pyrrolidone) (PVP), poly (N-isopropyl acrylamide) (PNIPAM), and poly (hydroxypropyl methacrylamide) (PHPMA) is widely used as shell-forming material. Nanomicelles have many advantages, including being structurally stable, having the ability to trap a large number of hydrophobic drugs, their surface can be conjugated with the targeting ligand [118], and they have a suitable size distribution to avoid rapid renal excretion, allowing accumulation into the tumor tissue through the effect of EPR [119].

In a study from A.F.A. Aisha et al., α-mangostin was prepared in PVP by solvent evaporation method and the intracellular delivery through endocytosis that may enhance the antitumor efficacy of α-mangostin was examined. The cellular uptake assay on human colon tumor cell line 116 (HCT16) showed high permeability of α-mangostin through the cytoplasmic membrane around the treated cells whereas nanomicelles showed fluorescence in the form of spherical particles. These results suggest that cellular uptake from nanomicelles can be mediated through endocytosis. The cytotoxic effect of α-mangostin and nanomicelles exhibited a significant cytotoxic effect in a dose-dependent manner with IC_50_ of 7.7 ± 0.1 and 8.9 ± 0.2 μg/mL, respectively. Cytotoxicity results show intracellular release of drug payload, which occurred due to the degradative effect of lysosomal enzymes and also show the interaction of PVP and α-mangostin does not affect α-mangostin’s cytotoxicity [43].

Another study of nanomicelles nanoformulation was used to improve the α-mangostin anti-melanoma effect. The α-mangostin/MPEG-PCL has a core structure with PCL (hydrophobic) which absorbs α-mangostin and MPEG (hydrophilic) as shells. The molecular modeling examined the interaction between the αM and MPEG-PCL as carriers which were observed by comparing the interactions in the aquatic environment and the tumor environment. The results showed that the interaction between α-mangostin and MPEG-PCL in an aqueous environment tended to persist with a spherical particle shape, whereas in the tumor environment, MPEG-PCL did not appear to be able to maintain their spherical particles so that it was easier for αM to exit the nanoparticle system. In in vitro cytotoxic testing, α-mangostin nanomicelles possessed a stronger inhibitory effect compared to the α-mangostin on A375 and B16 melanoma cells and exhibited low cytotoxicity for non-tumor cell lines (LO2, Vero, and HEK293T cells). The growth inhibitory effect of the α-mangostin and α-mangostin nanomicelles shows inhibition on melanoma cell colonies. Surprisingly, no colony formation was observed in A375 and B16 cells treated by α-mangostin nanomicelles. However, several colony formations under the same condition were formed in the α-mangostin group. These results suggest that α-mangostin nanomicelles have a more intense restraining effect on the colony formation rate in melanoma cells compared to α-mangostin. Moreover, the improved apoptotic activity of α-mangostin nanomicelles was determined compared with α-mangostin. It is because the nanomicelles had a better cellular uptake behavior than the α-mangostin. Furthermore, in vivo anticancer activity of the α-mangostin nanomicelles was observed using A375 cells injected subcutaneously into the female BALB/c athymic mice. The α-mangostin nanomicelles possessed a better effect on tumor growth inhibition (tumor weight and volume = 0.35 g and 400 mm^3^) compared to the free α-mangostin (0.7 g and 910 mm^3^). The α-mangostin nanomicelles held smaller tumors than α-mangostin. In conclusion, a strategy was presented in which the α-mangostin delivery system in nanomicelles comprehensively increases the anti-tumor activity of α-mangostin in vivo [80].

In another study, α-mangostin encapsulated with MPEG-PLA nanomicelles was formulated by S. Zheng et al. The nanomicelles show a spherical, monodisperse, and narrow particle size structure with an average size of 32 nm. In vitro antitumor activity of α-mangostin and nanomicelles on U87 cells indicate the inhibition of the proliferation process and promoted apoptosis. The antitumor effect of nanomicelles was higher when compared to α-mangostin, with apoptosis rates, at concentrations of 10 and 20 μg/mL, of 36.6% and 54.9% for nanomicelles and 20.4% and 45.5% for α-mangostin. Furthermore, the antitumor activity test on female C57/BL6 mice showed nanomicelles increased the antitumor effect of α-mangostin (65% reduction of tumor volume) and indicated by a decrease in tumor weight (0.62 g for nanomicelles and 1.14 g for α-mangostin), inhibited tumor growth, decreased proliferation, suppression of angiogenesis, and an increase in the apoptosis index that was two times higher than that of α-mangostin. This increase in activity is due to the particle size of nanomicelles at the nanoscale, being able to pass through the inter-endothelial junctions of a tumor by passive diffusion, and the use of PEG to increase the stability of nanoparticles in blood circulation and high retention in the tumor region [81].

### 4.7. Liposomal Nanoparticles

Liposomes are membrane vesicles composed of amphiphilic lipids that surround the water nucleus. Liposomes form spontaneously as lipid molecules are dispersed in a liquid medium, resulting in nanometer to micrometer size. Liposomes are formed by a phospholipid sheath composed of one or more lipid bilayers with hydrophilic head groups and hydrophobic tail groups [120,121]. This vesicle bilayer system enables the liposome to trap lipophilic and hydrophilic molecules, enabling these vesicles to bundle different drugs. Via the effect of increased permeability and preservation, encapsulation in the liposome structure can shield compounds from early inactivation, oxidation in the bloodstream, improve the half-life, and increase aggregation in the tumor [120,122,123].

R. Benjakul et al. developed liposomes nanoformulation and evaluated their cytotoxic effect and mechanism inducing cell death in various human carcinomas (human lung epithelial carcinoma (Calu-3), human colon carcinoma (HT-29), human breast carcinoma (MCF-7), and human colon carcinoma (Caco-2) cells). Liposome was prepared with phosphatidylcholine and cholesterol using the reverse-phase evaporation method. The particle size, polydispersity index, and zeta potential were 113.98 ± 2.95 nm, 0.13 ± 0.01, and −25.6 ± 0.07 mV, respectively. The cytotoxic effect trends of α-mangostin liposomes on human carcinoma cell lines revealed that α-mangostin liposomes were less toxic than α-mangostin. In all four measured cells, the IC_50_ was 2–4 times greater than α-mangostin. It is due to the disparity in the cellular absorption process, where α-mangostin can reach cells through passive diffusion, while α-mangostin liposomes can enter cells through adsorption, endocytosis, or the fusion mechanisms, then accompanied by an α-mangostin release from its carriers. Although free α-mangostin showed relatively higher toxicity than α-mangostin liposomes, the use of liposomes as nanocarriers can still be used as a promising α-mangostin delivery system for anticancer therapy to avoid direct toxicity of α-mangostin to normal cells (drug targeting) [82].

In another study from M.P. Nguyen Thi et al., α-mangostin liposomes were formulated with dioleoylphosphatidylcholine and cholesterol, and a cytotoxicity test was conducted on human hepatocellular carcinoma (Hep-G2) cells. There was a significant decrease in the IC_50_ value of 2.4 times between α-mangostin and α-mangostin liposomes (1.9 and 4.6 µM, respectively). Furthermore, the cell viability significantly reduced after 48 and 96 h in the α-mangostin liposome group compared with the α-mangostin. Due to the α-mangostin liposome group, α-mangostin is released slowly and the cells could absorb it gradually so that it kills fewer cells than free α-mangostin within 24 h and length of the period (48 and 96 h). It is consistent with the release of in vitro liposome that exhibits a sustained drug release profile. As for α-mangostin, cell viability varied with the concentration during the first 24 h. The results of this study indicate that liposomes exhibited a more effective cytotoxic effect against Hep-G2 cells as compared with free α-mangostin [83].

### 4.8. Gold Nanoparticles

Gold nanoparticles are excellent carrier molecules for cancer drug delivery. They can be synthesized in a variety of sizes and surface characteristics, which make them promising candidates as drug delivery vehicles. Multifunctional gold nanoparticles are now widely used in cancer therapy because of their inertness and biocompatibility. The surface of the gold nanoparticles can be easily modified to provide a controlled release strategy using internal or external stimuli [124,125,126]. S. Qiu et al. developed α-mangostin gold nanoparticles using gold citrate and coated with PEI and sulfated β-cyclodextrin to complex α-mangostin. This formula produces an average particle size of around 100 nm with a particle size distribution that is monodisperse. The zeta potential of the nanoparticles is 30 ± 3 mV, meaning that they are stable. In vitro cell viability assays of gold α-mangostin nanoparticles against prostate cancer cell lines (PC-3 and DU145) revealed a 15% and 50% improvement in activity relative to α-mangostin, respectively. Furthermore, α-mangostin nanoparticles are known to trigger an apoptotic death pathway that enables tumor cell damage and elimination through phagocytosis. According to the findings of this research, this drug delivery mechanism (gold nanoparticles) could increase the cytotoxicity of α-mangostin against prostate cancer cell lines [84].

## 5. The Perspective of the Authors

Recently, nanoformulations have been taken into consideration as a drug carrier as they have improved the pharmacokinetic properties of the drug to increase its efficiency and reduce side effects. In cancer treatment, targeted treatment only targeted the cancer cells to be killed and less harm to the normal cells is increasingly desirable. Nanotechnology has improved cancer therapy in many ways, such as selective recognition of cancer cells, targeted drug delivery, and overcoming the limitations of α-mangostin.

It is generally known that nanoparticles with smaller sizes and modified surface properties can reach or penetrate certain areas that normally cannot be passed through. Surface properties, particularly the surface charge of the particles, can also influence the distribution of the nanoparticles and possibly lead to higher toxicity in both cancer and non-cancer cells. Therefore, future research can perform surface functionalization of α-mangostin nanoformulations with specific antibodies, proteins, nucleic acids, and small molecules that can actively attach to cancer cells. Currently, CREKA peptide and thioaptamer are ligands that use α-mangostin nanoformulations. The use of these two ligands showed to increase the intracellular delivery and the efficacy of α-mangostin. There are many types of target ligands (such as antibodies and small molecules) that can develop in nanoformulations of α-mangostin to increase the anticancer effectiveness of α-mangostin.

Polymeric nanoformulation (PEG-PLA-CREKA), β-cyclodextrin nanoparticles, liposomes, gold nanoparticles, and nanomicelles (MPEG-PCL and MPEG-PLA) have particle size characteristics >150 nm, which are suitable for entry into cancer cells and increased cellular uptake of α-mangostin nanoparticles. Cytotoxicity testing in vitro also showed increased activity in the tested cancer cell lines, except for in β-cyclodextrin nanoparticles, there was a decrease in cytotoxicity in the CT26WT cell line and liposomes (phosphatidylcholine and cholesterol) in Calu-3, HT-29, MCF-7, and Caco-2 cell lines. It is due to differences in the cellular mechanism for uptake of free α-mangostin, which can pass into cells by passive transport (diffusion) mechanism, the β-cyclodextrin and liposome nanoformulations can be taken up into cells by adsorption and endocytosis and related to the α-mangostin release from the carrier. MPEG-PLA and MPEG-PCL nanomicelles were proved to improve the bioavailability of α-mangostin in mice which showed significantly reduced α-mangostin elimination. In addition, the in vivo anticancer activity showed better inhibition of tumor growth than α-mangosin.

Polymeric, fibroin, solid lipid nanoparticles are very promising to be developed due to biodegradable components, high-loading capacity (up to 17%), and easy preparation, but these formulas produce large particle sizes up to 400 nm. The development of nanoformulations for α-mangostin such as enteric coating nanoparticles (chitosan-kappa carrageenan) and mucoadhesive (NLC-CS) is an alternative for oral delivery of α-mangostin, by modifying the release stimulated by pH and the surface charge interactions of the nanoparticles that can target the release at specific areas of the gastrointestinal tract. Even though in vitro studies have been shown to increase cytotoxicity activity through the activity of inhibiting proliferation and increasing apoptosis against cancer cells, further studies regarding anticancer activity in vivo are needed. 

Although recent reports on α-mangostin nanoformulation to date appear promising, most of the published data come from in vitro and in vivo (Table 3) trials and not clinical trials. Therefore, there are concerns regarding the toxicity of nanoparticles, as little is known about the behavior of nanoparticles in humans. While the unique properties of nanoparticles brought about by their small size provide enormous opportunity for medicinal uses, safety concerns have surfaced since their physicochemical properties might lead to altered pharmacokinetics, with the ability to overcome biological barriers. Furthermore, the inherent toxicity of several of the compounds, as well as their capacity to collect and stay in the human body, has hampered their translation. The use of biological capping materials like such as chitosan further reduce toxicity while their biocompatibility and biodegradative capacity making them an intuitive choice for a nanocarrier. The stability, circulation time, access and bioavailability to disease locations, and safety profile of these nanoparticles are critical for successful clinical translation. Thus, clinical trials are ultimately needed to understand the in vivo behavior of the α-mangostin nanoformulation, which can ultimately lead to the design of a suitable formulation with superior therapeutic efficacy.

## 6. Conclusions

In recent years, various nanoformulations of α-mangostin have been developed to improve bioavailability and effectiveness in cancer treatment. α-mangostin exhibits excellent anticancer properties but its poor solubility, rapid elimination, and poor pharmacokinetic properties hinder its usability as a potent drug against cancer. Many techniques have been used to address this issue, one of which is the development of nanosized delivery vehicles. For α-mangostin formulated as nanoparticles, this development is very welcome because they are not only able to improve the dispersion of α-mangostin in aqueous solution, but also provide other advantages not obtained from conventional delivery techniques. These advantages include changeable particle size as well as modifiable surface characteristics. For example, the conjugation of α-mangostin nanoparticles with targeting ligands such as peptides and aptamers can provide specific targeting to cancer cells. With the nanoformulation, the researchers can improve the bioavailability and therapeutic properties of α-mangostin nanoparticles to achieve high therapeutic efficacy. Moreover, the α-mangostin nanoformulation appears to have good affinity and selectivity against cancer cells while imparting negligible toxicity to normal cells.

## Figures and Tables

**Table 1:** The physicochemical properties of α-mangostin [39,58].

Property	Description
Molecular formula	C_24_H_26_O_6_
IUPAC name	1,3,6-Trihydroxy-7-methoxy-2,8-bis(3-methylbut-2-en-1-yl)-9H-xanthen-9-one
Molecular weight	410.5
Color/Form	Faint yellow to yellow powder
Melting point	180–181 °C
Solubility	Soluble in methanol, in water (2.03 × 10^−4^ mg/L at 25 °C)
LogP	log Kow = 7.71
Stability	Stable under normal temperatures and pressures
Dissociation constants	pKa1 = 3.68 (primary carbonyl); pKa2 = 7.69 (secondary carbonyl); pKa3 = 9.06 (tertiary carbonyl)

**Table 2:** Summary of α-mangostin nanoformulations.

Carrier	Cell Line	Outcome	Ref.
Silk fibroin N-(3-Dimethylaminopropyl)-N′-ethylcarbodiimide hydrochloride (EDC) and polyethylenimine (crosslinker)	Caco-2 MCF-7	Increased solubility Sustained release Reduced hematotoxicity up to 90% Stable for up to 24 h when dispersed in IV diluent and 6 months when preserved as lyophilized powder at 4 °C Improved cytotoxicity and apoptosis in vitro	[3]
*Acetobacter xylinum*	B16F10; MCF-7; hGF; HaCaT	Significantly lower toxicity to normal cells than to cancer cells Slightly toxic to HaCaT cell (normal cells)	[70]
PLGA, soybean lecithin, DSPE–PEG2000–COOH Thioaptamer (ligand)	MCF-7	Particle size 150–300 nm Internalization of nanoparticles Strong disaggregation of MCF-7 multicellular tumor spheroids	[71]
Miglyol 812, cetyl paomitate, montanov 82, and oleoyl chitosan (coating agent)	Caco-2 Hela	Particle size < 200 nm High physical stability Excellent encapsulation efficiency (EE > 90%) High level cellular internalization Improved cytotoxicity Downregulation of cyclin D1-(CCND1) and anti-apoptotic gene BCL2	[72]
PLGA	Pancreatic cancer cell line (AsPC-1, PANC-1, and Mia-Paca-2) Cancer stem cells (CSCs)	Particle size < 200 nm Can easily enter into the cells Downregulation of pluripotency maintaining factors components of Shh pathway, Gli targets, EMT markers, transcription factors, and upregulation of E-cadherin Inhibit proliferation, colony formation; cell motility, migration, and invasion Induced apoptosis, inhibits the growth, inhibits development and metastasis of pancreatic cancer	[35]
PLGA	HCT116 and HT29 Normal epithelial cells (CRL-1831)	Internalization of nanoparticles Suppressing the expression of Notch receptors and their ligands, γ-secretase complex protein and downstream target Induced cancer cells and did not induce apoptosis in normal cells	[73]
α, β dan γ cyclodextrin (CD) Epichlorohydrid (ECH) as linker	CT26WT	The highest level of solubility and complexation efficiency of α-mangostin was shown in complexation with βCD CD nanoparticle α-mangostin complexes showed larger loading ratio than CDs themselves Rapid release and slow release Decreased cytotoxicity	[74,75]
β-cyclodextrin	A549	Particles size < 50 nm Nanoparticles taken up into the cancer cells and affected nuclear morphology Improved cytotoxicity	[76]
Chitosan and alginate Genipin as crosslinker	HT-29	Particle size around 400–500 nm Genipin as a crosslinker significantly increases the loading efficiency and loading capacity of the nanoparticles Improved cytotoxicity	[77]
Poly-(ethylene glycol)–poly(l-lactide) (PEG–PLA) CREKA peptide (ligand)	PANC-1 NIH3T3, PANC-1-Luc2	Particle size 100 nm Controlled and continuous release Increased intracellular delivery Suppressed NIH3T3 activation, decreased fibronectin expression (in vivo), promotes tumor vascular normalization and enhances blood perfusion (in vivo) Blocked TGF-β signaling pathways by inhibiting phosphorylated Smad2 and Smad3 protein synthesis (in vitro and in vivo); cancer-associated fibroblast (CAF) suppression and collagen downregulation effect	[78]
Chitosan and Kappa Carrageenan	MCF-7	Particle size 200–400 nm Excellent encapsulation efficiency (EE > 97%) Increased solubility Initial burst release Improved cytotoxicity	[79]
Polyvinylpyrrolidone (PVP)	HCT 116	Significantly increases the solubility of alpha mangostin (1000-fold) Particles size < 130 nm Nanomicelles can enter into cancer cell The half maximal inhibitory concentrations for α-mangostin nanomicelles was higher than raw α-mangostin	[43]
Monomethoxy poly (ethylene glycol)-polycaprolactones (MPEG-PCL)	A375 and B16 non-tumor cell lines (LO2, Vero, and HEK293T cells)	Mean particle size = 30 nm as a monodisperse system Drug loading up to 99.1% Sustained drug-release profile Excellent cellular uptake Induced apoptosis via the mitochondrial-mediated intrinsic pathway and the exogenous apoptosis pathway Pharmacokinetic study shows a slow excretion behavior from blood vessels	[80]
Methoxy poly(ethylene glycol)-poly(lactide) (MPEG-PLA)	U87	Mean particle size 32 nm and EE = 99.5% Sustained release Improved the pharmacokinetics of α-mangostin Suppression of protein bcl-2 decreased, pro-apoptotic protein Bax and cleaved-caspase-3, 8 and 9	[81]
Phosphatidylcholine and cholesterol	Calu-3; HT-29; MCF-7; Caco-2; HaCaT; HDF	Particle size around 100 nm Lower toxicity in normal cells than α-mangostin in aqueous solution Decreased cytotoxicity, induced apoptosis	[82]
Dioleoylphosphatidylcholine and cholesterol	Hep-G2	Particle size around 100 nm, high entrapment efficiency, slow and sustained release Improved cytotoxicity	[83]
Gold citrate	PC-3 DU145	Induced DNA fragmentation Improved cytotoxicity	[84]

**Table 3:** The volume reduction of tumor treatment with different α-mangostin nanoformulations.

Formulation	Type of Tumor	Reduction of Tumor Volume	Ref.
PLGA	Pancreatic	More than 60% of tumor reduction with 20 mg/kg dosage	[35]
Cyclodextrin nanoparticle	Colon	Approximately 56% of tumor reduction with 10 mg/kg dosage	[75]
PEG-PLA nanomicelles coated with CREKA peptide	Pancreatic	More than 70% of tumor reduction with 20 mg/kg dosage	[78]
MPEG-PCL nanomicelles	Melanoma	Almost 50% of tumor growth reduction with 50 mg/kg dosage	[80]
MPEG-PLA nanomicelles	Glioma	Approximately 65% tumor reduction with 50 mg/kg dosage	[81]

## Data Availability

Not applicable.
